# Interleukin-15 enhanced the survival of human γδT cells by regulating the expression of Mcl-1 in neuroblastoma

**DOI:** 10.1038/s41420-022-00942-5

**Published:** 2022-03-29

**Authors:** Hui Wang, Xiaolin Wang, Wei Wang, Wenjia Chai, Wenqi Song, Hui Zhang, Wenjun Mou, Mengmiao Pei, Yan Su, Xiaoli Ma, Jingang Gui

**Affiliations:** 1 Laboratory of Tumor Immunology, Beijing Pediatric Research Institute, Beijing Children’s Hospital, Capital Medical University, National Center for Children’s Health, Beijing, 100045 China; 2 Key Laboratory of Major Diseases in Children, Ministry of Education, Beijing Pediatric Research Institute, Beijing Children’s Hospital, Capital Medical University, National Center for Children’s Health, Beijing, 100045 China; 3 Department of Clinical Laboratory Center, Beijing Children’s Hospital, Capital Medical University, National Center for Children Health, Beijing, 100045 China; 4 Medical Oncology Department, Pediatric Oncology Center, Beijing Children’s Hospital, Capital Medical University, National Center for Children’s Health, Beijing Key Laboratory of Pediatric Hematology Ocology, Key Laboratory of Major Diseases in Children,Ministry of Education, Beijing, 100045 China

**Keywords:** Immune cell death, Cancer immunotherapy

## Abstract

Neuroblastoma (NB) is the most common extracranial solid tumor and the treatment efficacy of high-risk NB is unsatisfactory. γδT-cell-based adoptive cell transfer is a promising approach for high-risk NB treatment. Our previous study has revealed that γδT cells in NB patients exhibit a poor proliferation activity and a decreased anti-tumor capacity in vitro. In the present study, we found that IL-15 could effectively enhance the proliferation of NB γδT cells, to a level that remains lower than healthy controls though. In addition, IL-15-fostered NB γδT cells robustly boosted cell survival against apoptosis induced by cytokines depletion. Our data revealed that Mcl-1 was a key anti-apoptotic protein in IL-15-fostered γδT cells during cytokine withdrawal and its expression was regulated via the activation of STAT5 and ERK. In addition, IL-2 and IL-15-fostered γδT cells harbored higher levels of tumoricidal capacity which is also beneficial for γδ T-cell based immune therapy in NB. Understanding the survival control of γδT cells in a sub-optimal cytokine supportive microenvironment will expedite the clinical application of γδT cells for immunotherapy.

## Introduction

Neuroblastoma (NB) is the most common extracranial solid tumor that accounts for 8% of childhood cancers [1]. At present, ~40–50% of NB patients are diagnosed with high-risk NB with an overall survival rate <50% owing to high recurrence and metastasis, which cannot be curtained by traditional surgery and chemo/radiotherapy [2–4]. Novel strategies such as immunotherapy are eager to be developed for tackling the devastating NB in children.

Adoptive transfer of γδT cells holds great promise for immunotherapy [5], thanks to their strong anti-tumor activity, readily expansion, and independence of major histocompatibility complex molecules as opposed to αβT cells. It has been documented that in vitro expanded γδT cells showed clinically relevant cytotoxicity to NB cells [6]. However, the production of γδT cells stimulated with IL-2 in vivo was shown to concurrently promote the generation of regulatory T cells, potentially inhibiting immune surveillance for cancer cells [7]. Our previous study also demonstrated that in vitro expanded NB-derived γδT cells featured with a reduced proliferation capacity compared with expanded γδT cells from healthy controls (HC) [8]. Previous research revealed that IL-15 could promote cell proliferation, the anti-tumor function of γδT cells, and enhance the response of γδT cells to microbial pathogens [9]. Therefore, it is valuable to test whether IL-15 can rectify and promote subdued NB-derived γδT cells to regain better survivability with a reinforced proliferation potential competent for autogenic γδT adoptive transfer.

In the clinical setting, adoptive transfer therapy of γδT cells requires harvesting enough cells for engraftment, and sustaining cell survival and cytotoxicity lasting long enough for tumor eradication [10]. Although supraphysiological levels of cytokines were added in vitro culture to support γδT-cell expansion, cytokines with a level out of scope were not appropriate to administrate directly to patients due to potentially severe side effects [11]. In vitro expanded γδT cells prepared in a condition supplemented with high concentrations of IL-2 and IL-15 would abruptly dive into a survive niche with a physiological level of cytokines in the recipient host far below the culture ones upon adoptive transfer. The drastic environmental change in vivo could affect the survival and function of these well-behaved immunocompetent cells prepared in vitro. For instance, the proliferation of NK cells displayed a dose-dependent addiction to IL-15 and the sudden withdrawal of IL-15 increased the apoptosis with altered expression of Bcl-2 family proteins [12, 13]. The stronger survival and cytolytic activity were observed in IL-15-primed NK cells attributed to a STAT5-dependent upregulation of Bcl-2 [14]. Anti-apoptotic Bcl-2 family members including Bcl-2 and Mcl-1 express in different cells and play critical roles in regulating their life and death through the intrinsic death pathway [15]. Mcl-1, a short half-life anti-apoptotic protein, undergoes rapid upregulation and stabilization in response to cytokines or antigen signaling. IL-15, as a pro-survival cytokine, regulates the expression of Bcl-2 family members to maintain the survival of T cells [12]. However, it is unclear if the survival of γδT cells is regulated by IL-15 via the expression of Bcl-2 family proteins, and what is the destiny of expanded IL-15-primed γδT cells in a system devoid of cytokines.

In the present study, we aimed to enhance the survival of γδT cells for adoptive cell transfer therapy by optimizing the previous in vitro cell-expanding protocol and further explore the maintenance of γδT-cell survival in NB patients receiving immunotherapy. We found that IL-15 effectively promoted the proliferation of γδT cells and robustly increased apoptotic resistance when cytokines were suddenly depleted. We fathomed the underlined factors involved in the cell protection and found it was Mcl-1 but not Bcl-2 that promoted the cell longevity after IL-15 withdrawal, a process mimicking the abrupt change of cytokine levels when in vitro expanded cells are adoptively transferred to patients. In addition, the level of Mcl-1 was regulated by the activation of STAT5 and ERK in IL-15-fostered γδT cells. Notably, regardless of patient- or HC-derived preparation, IL-15-fostered γδT cells co-cultured with NB cell line exhibited an increased cytolytic activity. Results from our study suggested that in vitro expanding NB patient-derived γδT cells requires IL-15 nourishment to transform into apoptosis-resistant immunocompetent cells optimized for autologous adoptive cell transfer therapy.

## Results

### IL-15 robustly promoted γδT-cell proliferation during in vitro culture

**Fig. 1:**
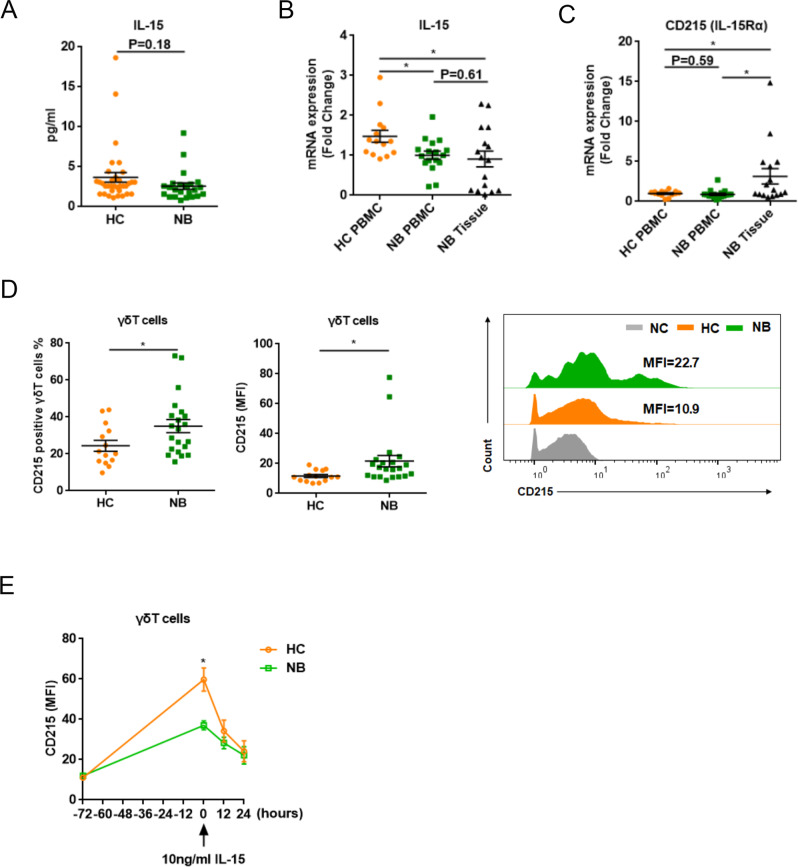
Qualification of IL-15 and IL-15Rα in NB patients. **A** IL-15 levels in plasma were analyzed by ELISA (HC, *n* = 34; NB, *n* = 23). Data were mean ± SEM and statistical analysis was performed using an unpaired *t* test. **B**, **C** The expression of IL-15 and CD215 (IL-15Rα) were determined by qPCR (HC, *n* = 14; NB, *n* = 33, including 17 of NB PBMCs and 16 of NB tissues). Data were mean ± SEM and statistical analysis was performed using one-way ANOVA and Dunnett’s multiple comparisons. *, p < 0.05. **D** The expression of CD215 in γδT cells was analyzed by flow cytometry (HC, *n* = 14; NB, *n* = 21). Data were mean ± SEM and statistical analysis was performed using an unpaired *t* test. *, p < 0.05. **E** The expression of CD215 in γδT cells was induced for 72 h and then IL-15 was added for another 24 h. The expression of CD215 was analyzed by flow cytometry at each time point. (HC, *n* = 5; NB, *n* = 5). Statistical analysis was performed using an unpaired *t* test. *, p < 0.05. NC negative control; HC healthy controls; NB neuroblastoma patients.

We previously have known that γδT cells in NB patients presented with a reduced proliferation capacity and diminished cytotoxicity against tumor cells [8]. Considering the crucial role of IL-15 in the maintenance of long-lasting, high-avidity T-cell responses [16], our speculation was that the insufficiency of IL-15 concentration in patients was possibly one of the adverse factors for the survival and function of γδT cells. By ELISA method, we found the serum concentration of IL-15 slightly decreased in NB patients (Fig. 1A). This decrease was subsequently verified by quantitative PCR (qPCR) taking templates out of from patient blood and tumor tissues (Fig. 1B). Conversely, CD215, the alpha subunit of IL-15 receptor (IL-15Rα), was found to express a higher level in NB tissues as well as in NB-derived γδT cells (Fig. 1C, D). In an in vitro setting, the expression of IL-15Rα on HC γδT cells was much higher than it was on the patient γδT cells in a 3-day expanding culture with IL-2 only. Nevertheless, the attrition of IL-15Rα in patient γδT cells triggered by the addition of IL-15 in culture after cell expanding was at a slower rate in a 24 h observation time window suggesting dull signaling transduction (Fig. 1E).

**Fig. 2:**
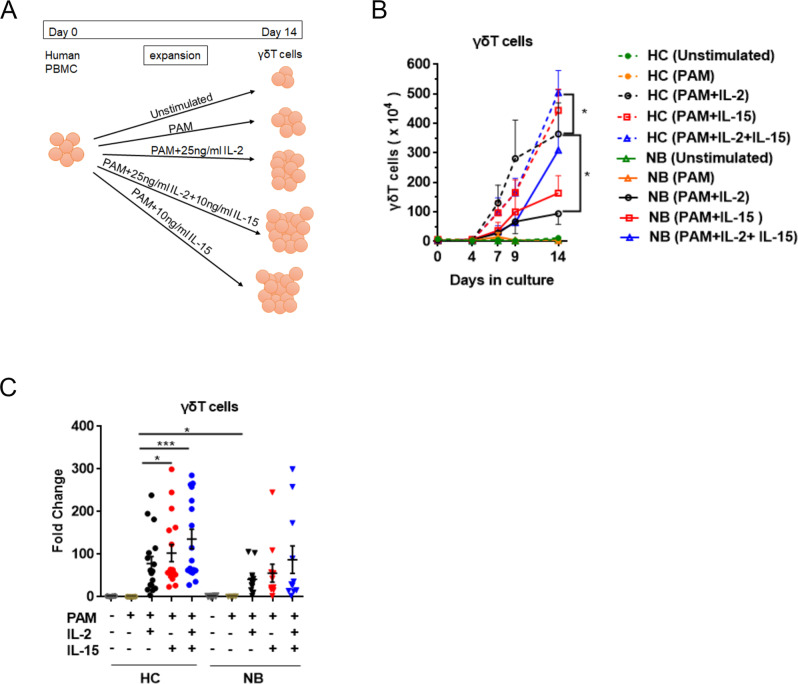
Qualification of γδT-cell expansion capacity in vitro culture conditions. γδT cells were stimulated with PAM supplemented with either IL-2 or IL-15 or both of them and expanded in vitro for 14 days. **A** Flowchart of the experimental setup to study in vitro γδT-cell expansion capacity with different culture conditions. **B** At culture days 0, 4, 7, 9, and 14, the absolute numbers of γδT cells from HC and NB were measured by flow cytometry (HC, *n* = 7; NB, *n* = 7). Data were mean ± SEM from four independent experiments. Statistical analysis was performed using one-way ANOVA and Dunnett’s multiple comparisons. *, p < 0.05. **C** fold change was calculated by dividing the number of γδT cells on day 14 by the numbers of γδT cells at the start (HC, *n* = 17; NB, *n* = 11). Data were mean ± SEM from five independent experiments. Statistical analysis was performed using one-way ANOVA and Dunnett’s multiple comparisons. *, p < 0.05; ***, p < 0.001.

To further investigate the efficacy of IL-15 for fostering the proper proliferation and tumoricidal activity of in vitro expanded human γδT cells. We cultured the γδT cells from patients and healthy controls in the presence of stimulant PAM (pamidronate) supplemented with either IL-2, or IL-15, or both of them. Unstimulated γδT cells were not able to proliferate and were used as a negative control in this experiment (Fig. S1A). Following 4 days, 7 days, 9 days, and 14 days of culture, the percentage of γδT cells was determined by flow cytometry. Results showed a remarkable increase in the percentage of γδT cells along with the time of culture, but no differential percentages of γδT cells amongst the three culture conditions were observed in any of the time points we sampled (data not shown). To be noted, both NB- and HC-derived γδT cells achieved a much higher absolute cell number in the presence of IL-2 and IL-15 at the end of the 14-day expanding culture (Fig. 2B). Meanwhile, a greater fold change was also observed in IL-2 and IL-15 co-fostered γδT cells compared with IL-2 or IL-15 alone (Fig. 2C). Though IL-2 and IL-15 could robustly promote γδT-cell proliferation, NB-derived γδT cells exhibited a lower expansion capacity than HC with the IL-2 and IL-15 culture condition (Fig. 2B). On day 9, γδT cells from NB patients entered a fast-proliferating stage while it occurred as early as the fourth day in the culture of HC-derived γδT cells. Furthermore, with IL-2 and IL-15 culture conditions, NB-derived γδT cells achieved 3.09 ± 2.04 × 10^6^ cells compared with 5.04 ± 7.93×10^6^ cells from HC-derived γδT cells at the end of 14-day culture. In other words, IL-15 together with IL-2 could effectively enhance the proliferation of NB-derived γδT cells, to a level remaining lower than that of HC (Fig. 2C).

### IL-15-fostered γδT cells exhibited a stronger resistance to apoptosis after cytokine withdrawal

**Fig. 3:**
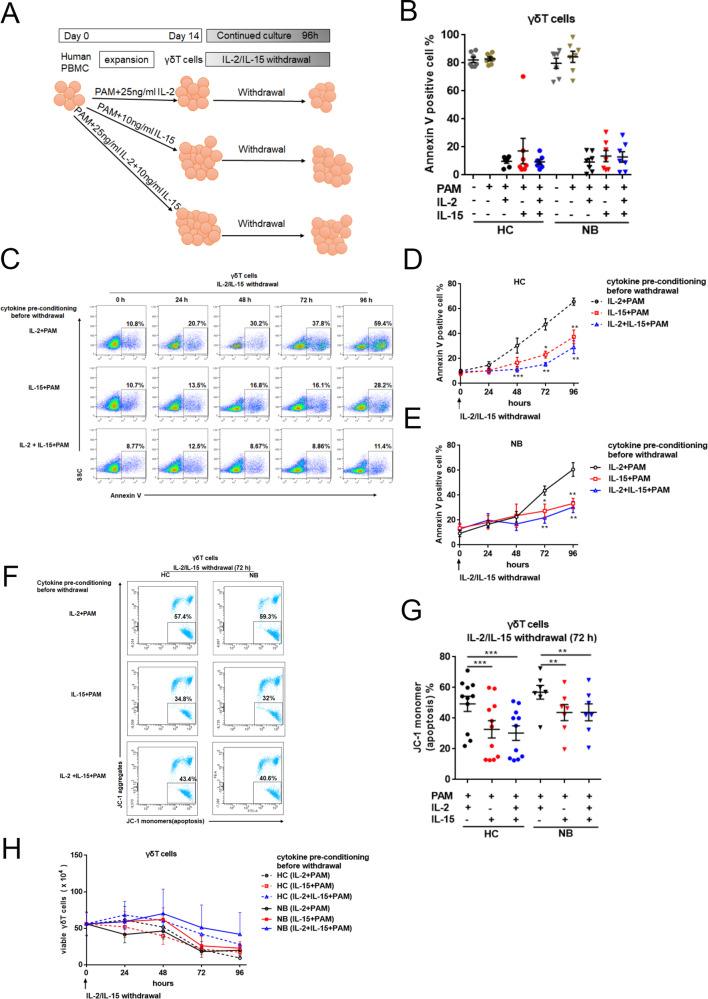
Qualification of γδT-cell apoptotic resistance after cytokine withdrawal. When γδT cells were cultured with IL-2, IL-15, and IL-2 plus IL-15 for 14 days, cytokines were removed and apoptosis was analyzed by measuring the level of Annexin V and 7-AAD by flow cytometry. **A** Flowchart of the experimental setup to study effects of IL-15 stimulation and withdrawal in γδT cells. **B** Annexin V^+^ γδT cells were analyzed at day 14 (HC, *n* = 7; NB, *n* = 7). Data were mean ± SEM from four independent experiments. Statistical analysis was performed using one-way ANOVA and Dunnett’s multiple comparisons. **C** Representative gating strategy for the flow-cytometric analysis. γδT cells fostered with different conditions were examined after cytokine withdrawal 0 h, 24 h, 48 h, 72 h, 96 h. **D**, **E** the proportion of Annexin V^+^ γδT cells were analyzed at each time point after cytokine withdrawal (HC, n = 7; NB, n = 7). Data were mean ± SEM from four independent experiments. Statistical analysis was performed using one-way ANOVA and Dunnett’s multiple comparisons. *, *p* < 0.05; **, *p* < 0.01; ***, *p* < 0.001. **F** Representative gating strategy for the flow-cytometric analysis. Mitochondrial membrane potential was analyzed by flow cytometry following staining with JC-1 when cytokines were withdrawn for 72 h. JC-1 aggregates are representative of high mitochondrial membrane potential and JC-1 monomers are representative of low mitochondrial membrane potential (apoptosis). **G** JC-1 monomer percentage was analyzed (HC, *n* = 11; NB, *n* = 7). Data were mean ± SEM from six independent experiments. Statistical analysis was performed using one-way ANOVA and Dunnett’s multiple comparisons. **, *p* < 0.01; ***, p < 0.001. **H** Annexin V^-^ 7-AAD^-^ γδT cells represented viable cells were assessed (HC, *n* = 7; NB, *n* = 7). Data were mean ± SEM from four independent experiments. Statistical analysis was performed using one-way ANOVA and Dunnett’s multiple comparisons.

Under physiological conditions, IL-2 and IL-15 were at an extremely low level especially in NB patients (IL-2 0.44 ± 0.05 pg/ml in HC vs 0.27 ± 0.02 pg/ml in NB patients, *p* < 0.05; and IL-15 3.63 ± 0.6 pg/ml in HC vs 2.54 ± 0.39 pg/ml in NB patients, *p* = 0.18, respectively). To mimic physiological conditions, cytokines were withdrawn when γδT cells were successfully expanded, and cell apoptosis was measured by Annexin V expression at various time points within the additional 96 h incubation (Fig. 3A). As previously mentioned, cultures without stimulants could not proliferate and encountered massive apoptosis (~80% of Annexin V^+^ γδT cells), so did the cultures have stimulants but without any cytokine support. With PAM stimulation, no evident apoptosis was observed in Petri dishes supplemented with IL-2 or/and IL-15 on a 14-day cell-expanding culture (Fig. 3B). No matter they originated from healthy controls or patients, in the following 96 h culture without cytokines, γδT cells initialized apoptosis with an increment over culture time as detected by Annexin V staining (Fig. 3C–E). The divergence of cell survival protection in the cultures of different cytokine pre-conditioning became obvious at 72 h sampling time after cytokine withdrawal, while the difference was observed as early as 48 h in the culture of HC-derived γδT cells (Fig. 3C–E). Based on our data, the cell survival protection after cytokine withdrawal was closely relevant to IL-15, as γδT cells expanded from IL-15 or IL-15 plus IL-2 pre-conditioning perceivably exhibited a decreased rate of cell death in a culture without further cytokine support (Fig. 3C–E). After 96 h cytokine-free culture time, ~60% of cells pre-conditioned only with IL-2 died (66 ± 5.08% in HC and 60 ± 4.57% in NB patients). By contrast, only 30% of those cells pre-conditioned with IL-15 or IL-15 plus IL-2 underwent apoptosis (30.5 ± 4.97% in HC and 24 ± 5.64% in NB patients) (Fig. 3D, E). To count the total viable cells in the Petri dishes after 96 h culture devoid of cytokine, we found starting from a cell seeding number 5 × 10^5^, cells pre-conditioned with IL-15 or IL-15 plus IL-2 outnumbered cells pre-conditioned only with IL-2. This trend was more obvious in the IL-15 plus IL-2 pre-conditioning group, suggesting a possible synergic effect from these two cytokines in cell protection during abrupt fluctuation of cytokine concentration (Fig. 3H).

Mitochondrial membrane potential (MMP) is a key indicator and initiator of cell apoptosis [17]. We used JC-1 dye to detect the loss of MMP 72 h post-cytokines withdrawal, at which massive apoptotic events occurred. As expected, JC-1 staining verified that γδT cells pre-conditioned with IL-2 showed a decreased aggregate to monomer ratio indicating that change in MPP was more obvious than in other cultures. Our JC-1 staining data revealed that IL-15 and IL-2 plus IL-15 fostered γδT cells exhibited a similar level of MPP change in cultures starting from both HC and NB Peripheral blood mononuclear cells (PBMCs) (Fig. 3F, G). To be noted, in any culture supplemented with IL-15 as pre-conditioning cytokine, a better survival rate was achieved in comparison with that supplemented with IL-2 only (Fig. 3C–H). These data revealed a more crucial role of IL-15 in protecting γδT cells from cell death during cytokines withdrawal. This underscores that IL-15 would be an indispensable factor for in vitro preparation of immunocompetent γδT cells with endurable survivability opt for immunotherapy, particularly for the autologous cell preparation where the fitness of starting cells is already an issue.

### IL-15-fostered γδT cells possessed stronger survival capacity through retaining the expression of Mcl-1 but not Bcl-2

**Fig. 4:**
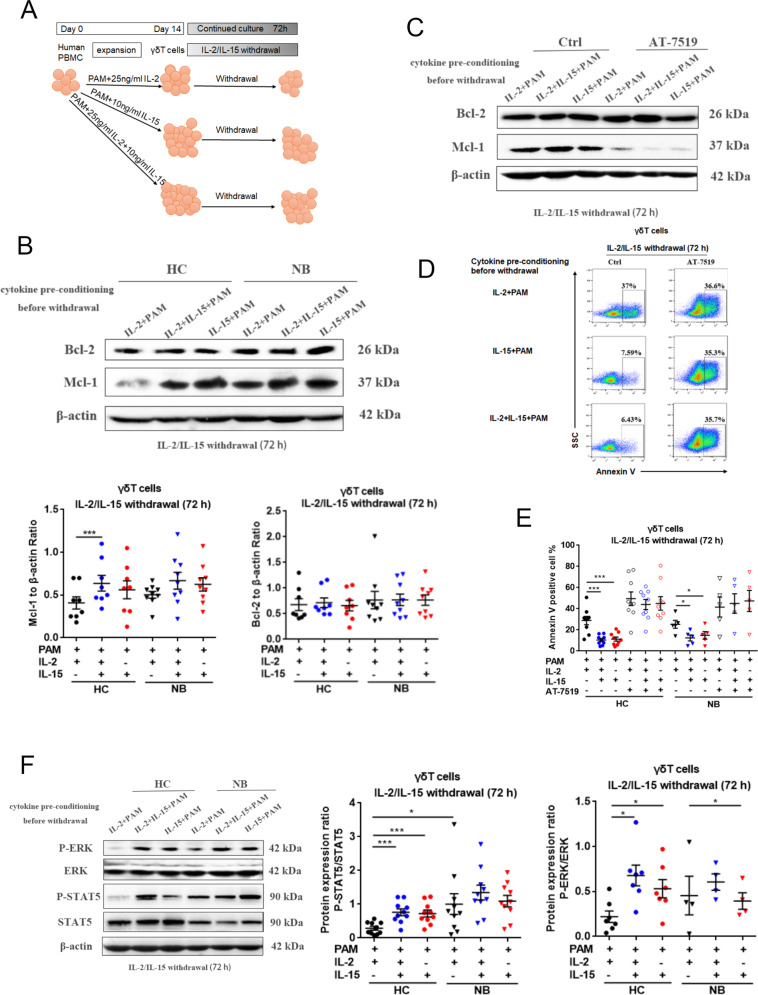
Mechanism of the enhanced survivability of γδT cells under IL-15 culture condition. When γδT cells were cultured for 14 days, cytokines were withdrawn for 72 h and γδT cells were collected and analyzed. IL-2-fostered γδT cells as a control culture condition. **A** Flowchart of the experimental setup to study potential the mechanism of IL-15 to enhance the survivability of γδT cells. **B** Western blotting was performed to analyze the expression of Mcl-1 and Bcl-2 (HC, *n* = 8; NB, *n* = 9). Data were mean ± SEM from five independent experiments. Statistical analysis was performed using one-way ANOVA and Dunnett’s multiple comparisons. ***, *p* < 0.001. **C**, **D** γδT cells were treated with AT-7519 for 24 h and representative results were shown for the Western blotting and the flow-cytometric analysis. **E** Annexin V^+^ γδT cells were analyzed when γδT cells were treated with AT-7519 for 24 h (HC, *n* = 11; NB, *n* = 5). Data were mean ± SEM from three independent experiments. Statistical analysis was performed using one-way ANOVA and Dunnett’s multiple comparisons. *, *p* < 0.05; ***, *p* < 0.001. **F** Western blotting was performed to analyze the expression of STAT5 and ERK and the phosphorylation levels of STAT5 and ERK. The protein band intensity was quantified and analyzed by ImageJ (HC, *n* = 8; NB, *n* = 9). Data were mean ± SEM from four independent experiments. Statistical analysis was performed using one-way ANOVA and Dunnett’s multiple comparisons. *, *p* < 0.05; ***, p < 0.001.

The homeostasis of T cells is strictly maintained by the up- and downregulation of Bcl-2 family members [18]. To clarify whether Bcl-2 family protein participates in the apoptosis resistance of IL-15-fostered γδT cells, we monitored the expression of Bcl-2 and Mcl-1 in γδT cells in cultures devoid of pre-conditioning cytokines (Fig. 4A). At 72 h post cytokine removal, the protein expression of Mcl-1 but not Bcl-2 was largely retained in γδT cells from culture pre-conditioned with both IL-15 and IL-2, in contrast to the attrition of Mcl-1 expression observed in γδT cells in culture pre-conditioned with IL-2 only (Fig. 4B). The Mcl-1 expression was retained in cultures pre-conditioned only with IL-15 as well in comparison with those pre-conditioned with IL-2 only (*p* = 0.06), the expression level of Mcl-1 was in accordance with the apoptotic rate of γδT cells continued to culture in the medium without any cytokines. The IL-15 pre-conditioned cultures exhibited an apoptotic rate around 23.16 ± 4.7% in HC and 27.07 ± 4.01% in NB patients, while the cultures pre-conditioned only with IL-2 presented with a ratio of 47.46 ± 4.54% in HC and 43.66 ± 3.61% in NB patients. Taken together, these data suggested that IL-15 has a protective effect on the γδT-cell survivability through sustaining the Mcl-1 expression in an environment scarce of cytokines.

To further verify the dependence of Mcl-1 in resisting apoptosis of γδT cells deprived of cytokine support, AT-7519, a specific inhibitor of Mcl-1, but not Bcl-2, was added to cultures 48 h post cytokine removal [19]. As expected, the expression of Mcl-1 but not Bcl-2 was entirely inhibited by AT-7519 at the time point of 72 h post cytokine removal in comparison with cultures treated with DMSO as control (Fig. 4C). Accordingly, γδT cells in cultures with AT-7519 indistinguishably underwent massive apoptosis in all culture groups regardless of what cytokines were added before the cytokine-free culture (Fig. 4D, E). These data indicated that the inhibition of Mcl-1 abolished the IL-15-mediated anti-apoptotic effect on in vitro expanded γδT cells.

STAT5 is well recognized as a common downstream pathway from cytokines receptors [20]. The expression of anti-apoptotic proteins Mcl-1 and Bim were upregulated by constitutively activated STAT5 [21]. According to our findings, to further investigate whether JAK/STAT pathway is involved in the Mcl-1 regulation in γδT cells receiving IL-15 nourishment, the phosphorylation of STAT5 was analyzed. After 72 h cytokine-free culture, it was perceivable that the phosphorylation of STAT5 in cultures pre-conditioned with IL-15 or IL-15 plus IL-2 was at a greater level than cells in cultures pre-conditioned only with IL-2 (Fig. 4F). Interestingly, the NB-derived γδT cells had a grossly higher level of phosphorylated STAT5 after 72 h cytokine removal in any pre-conditioned cultures. IL-15 is also known to stimulate MAPK/ERK pathways in addition to the JAK/STAT pathway [20]. Our findings indicated that the MAPK/ERK pathway was activated as phosphorylation of ERK became higher in control-derived γδT cells from IL-15 pre-conditioned cultures, but unexpectedly not in respective patient-derived γδT cells cultures.

### IL-15-fostered γδT cells increased the expression of activated phenotype and promoted the cytotoxic activity

**Fig. 5:**
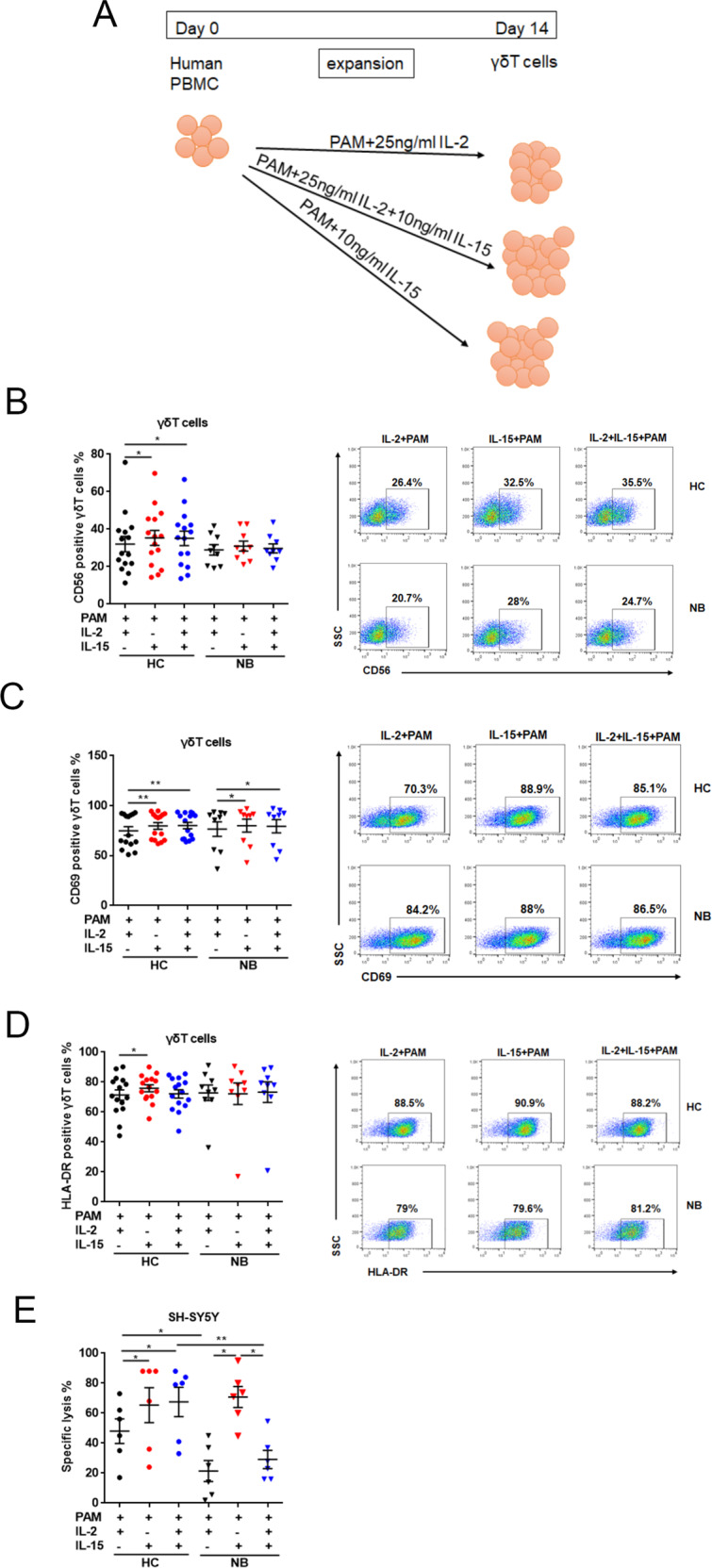
Qualification of γδT-cell activated phenotype and cytolytic activity. **A** Flowchart of the experimental setup to study activated phenotype and cytolytic activity of IL-15-fostered γδT cells. **B**–**D** The expression of activated markers including CD69, CD56, and HLA-DR in expanded γδT cells was measured by flow cytometry at day 14 (HC, *n* = 15; NB, *n* = 9). Data were mean ± SEM from three independent experiments. Statistical analysis was performed using an unpaired *t* test. *, p < 0.05; **, *p* < 0.01. **E** The cytotoxic assays were performed and expanded γδ T cells were cultured with NB cell line SH-SY5Y at the ratio of 1:10 (HC, *n* = 6; NB, *n* = 6). Data were mean ± SEM from three independent experiments. Statistical analysis was performed using one-way ANOVA and Dunnett’s multiple comparisons. *, *p* < 0.05; **, *p* < 0.01.

Now that we knew IL-15 could enhance the viability of γδT cells, we were curious about their functionality. To assess this, we first performed the flow cytometry staining to measure the expression of activating markers on the γδT-cell surface. Expression of HLA-DR, CD56, and CD69 in the 14-day in vitro expanded control-derived γδT cells were remarkably upregulated in cultures supplemented with IL-15 related to those cultured with IL-2 only. In contrast to control-derived γδT cells, in vitro expanded patient-derived γδT cells were not much affected except that the expression of CD69 was boosted by the addition of IL-15 in cultures (Fig. 5B–D). That being said, the cytotoxicity of patient-derived γδT cells against target tumor cell SH-SY5Y was definitely enhanced in the presence of IL-15 only, compared to those cultured in IL-2 only. The IL-2 plus IL-15 culture, however, by unknown mechanism abolished the boost effect from IL-15. While the control-derived γδT cells cultured in IL-2 only have a better tumoricidal capability than patient-derived cells in the same culture condition, both IL-15 only and IL-15 plus IL-2 culture boosted the target cell killing ability in relation to culture with IL-2 only as baseline (Fig. 5E).

## Discussion

γδT-cell immunotherapy is a promising treatment for NB patients which requires a large number of γδT cells preparation [22, 23]. Numerous studies have focused on expanding γδT cells using phosphoantigens (such as PAM) in the presence of IL-2 as a co-stimulator [11, 24]. As expected, the unstimulated γδT cells encountered massive apoptosis after a 14-day culture. While IL-15 shares many functions with IL-2, it was proved to promote the homeostatic expansion of mouse γδT cells in vivo and to inhibit the growth of αβT cells which expedite the expansion of γδT cells [25, 26]. This is crucial for tumor immunotherapy since the infiltration of γδT cells in most tumors is inefficient in comparison to αβT cells. The former, however, in most cases is functionally more tumoricidal. In our study, we successfully produced more γδT cells in vitro by adding IL-15 in the culture for both HC-derived and NB-derived PBMCs. In the end-stage of cell expansion, neither cultures with different cytokine supplements nor cultures from different cell origins of seeding (HC vs NB) were found different in terms of cell apoptosis. This implied that IL-15 could enhance the proliferation of γδT cells during in vitro expansion by PAM. That being said, even with the IL-15 supplement, the total number of γδT cells generated from culture seeded with patient-derived PBMCs was not able to reach the level of those started from HC-derived PBMCs. One of the reasons for curtailed maximum expansion in patient-derived γδT cells could be the dull responsiveness of IL-15Rα to IL-15 resulting from the long-term deficiency in IL-15 within the tumor microenvironment. Our data indicated that in NB patients, the expression of IL-15Rα in γδT cells was significantly higher than that from HC. This might be a salvage compensation to the sub-physiological level of IL-15 in the patient with a chronic tumor burden. The internalization of IL-15/IL-15Rα on NK cells modulated cellular response and IL-15Rα shedding following IL-15 stimulant was crucial to T-cell proliferation [27, 28]. Unexpectedly, reduction of IL-15Rα with a slower rate was observed in patient γδT cells triggered by the addition of IL-15, suggesting a sluggish IL-15/IL-15Rα complex internalization or shedding. We speculated that it was the desensitized IL-15/IL-15Ra signaling that led to a sub-optimized in vitro expansion of patient γδT cells. Whether and, if any, how the IL-15/IL-15Ra signaling pathway was attenuated requires further investigation.

It has been evidenced that absence of IL-15 in ~30% of patients with metastatic colon cancer was associated with high disease relapse and mortality [29]. In an in vitro experiment, IL-15-fostered NK cells maintained a lower level of apoptosis and a higher level of cytotoxicity after cytokine withdrawal compared with IL-2-fostered cells [20]. In our study, by mimicking the in vivo tumor microenvironment, we depleted the IL-15 after the 14-day cell expansion and found the patient-derived γδT cells possessed a much stronger anti-apoptotic ability in the following 96 h culture. We boldly believed that γδT cells in NB patients bore a tolerance to the extremely low level of IL-15 within the tumor microenvironment.

IL-15 regulated the apoptosis of γδT cells mainly through the intrinsic apoptosis pathway, a pathway similarily delayed human neutrophil apoptosis by Mcl-1 [30]. Our data clarified that IL-15 protected γδT cells from scarce of cytokines through sustaining the Mcl-1 expression, and the effect could be reversed by Mcl-1 inhibition with a CDK inhibitor (AT-7519). It has been reported that IL-15 failed to increase the level of Mcl-1 mRNA but changed its proteasomal degradation. Furthermore, IL-15 regulated Mcl-1 through JAK/STAT and PI3K/AKT pathways in T cells [21, 31]. As we discovered, the phosphorylation of STAT5 and ERK were dramatically changed in IL-15-fostered γδT cells. It is very likely that the anti-apoptotic effect in γδT cells conferred by IL-15 was through retaining the Mcl-1 via JAK/STAT5 or MAPK/ERK pathways.

Our results showed that the activation markers CD56, CD69, and HLA-DR were significantly upregulated in IL-15-expanded γδT cells on day-14 culture. This indicated that IL-15-fostered γδT cells exhibited an increased cytotoxic capacity, as confirmed by the promoted killing of NB cell line SH-SY5Y in our research. These results were consistent with the previous study that IL-15^−/−^ knockout mice only produced less amounts of IFN-γ upon stimulation and showed remarkably attenuated cytotoxicity against target cells as compared to wild-type mice [32]. Interestingly, in our study, the NB γδT cells supposedly having dull responsiveness to IL-15 signaling was still capable of killing target tumor cells, in a level similar to that from HC γδT cells. It reinforced our confidence in the usage of IL-15 pre-conditioned patient-derived γδT cells in an autologous adoptive transfer setting for tumor eradication. Furthermore, many hurdles, such as γδT-cell exhaustion, restricted trafficking, and limited tumor infiltration should be considered before clinical application.

## Materials and methods

### Sample collection

A total of 63 patients (mean age 4.2 ± 0.5 years) with NB at intermediate (13 boys and 7 girls) and high (25 boys and 18 girls) risk stages, and 58 healthy children (30 boys, 28 girls; mean age 3.9 ± 0.4 years) under regular physical examination were recruited from the Beijing Children’s Hospital Affiliated to the Capital Medical University. The study was approved by the Medical Ethics Committee of Beijing Children’s Hospital, the Capital Medical University. Written consent for research purposes of sample use was read and signed by all participants or their biological parents or legal guardians. Tumor samples from 16 NB patients were collected for this study. Peripheral blood samples from healthy children and NB patients were collected in BD Vacutainer™ plastic blood collection tubes with ethylenediaminetetraacetic acid (EDTA)-K2 as an anticoagulant during the onset of the disease. Serum samples were collected in tubes without anticoagulant by centrifugation at 600 × *g* for 5 min and the aqueous phase was taken.

### PBMC isolation

PBMCs were isolated from EDTA anticoagulated blood with Ficoll-Hypaque solution by density gradient centrifugation. After centrifuged at 1000 × *g* for 20 min at room temperature (RT), the interphase layer was carefully transferred into a 15 ml falcon tube with 10 ml PBS. PBMCs were obtained with another centrifugation at 600 × *g* for 5 min and resuspended in Roswell Park Memorial Institute (RPMI)-1640 (Gibco, Invitrogen, Carlsbad, CA, USA) supplemented with 10% FBS (fetal bovine serum, Gibco, Invitrogen, Carlsbad, CA, USA).

### NB cell line cultures

The NB cell line SH-SY5Y (CRL-2266) was purchased from American Type Culture Collection (Manassas, VA, USA) and cultured in DMEM (Gibco, Invitrogen, Carlsbad, CA, USA) supplemented with 10% FBS at 37 °C in 5% CO_2_.

### γδT-cell expansion

In order to induce activation of γδT cells, PBMCs were incubated with 10 µg/ml PAM (Sigma-Aldrich, St Louis, USA) in 10% FBS RPMI-1640 plus 25 ng/ml recombinant human IL-2 (Peprotech, Rehovot, Israel) and/or 10 ng/ml recombinant human IL-15 (Peprotech, Rehovot, Israel) at 37 °C humidified cell incubator with 5% CO_2_. Half of the culture medium was replaced by a fresh medium and recombinant cytokines were added every 3 days. The purity of γδT cells was examined on days 0, 4, 7, 9, and 14 of culture by flow cytometry analysis. Then cell counting was determined by Precision Count Beads™ (BioLegend, San Diego, CA, USA). Only those cells under a 14-day culture presented with a ratio >90% γδ-TCR and CD3-positive cells were considered as a successful expansion and pure enough for further experiments.

For some cultures, γδT cells were pre-treated with AT-7519 (Selleck Chemicals, Houston, TX, USA) for 24 h before further culture in a regular culture medium devoid of cytokines. Cells were then harvested for corresponding assays.

### Apoptosis analysis by Annexin V surface exposure

To determine the viability of in vitro expanded γδT cells in different cytokine-conditioned culture medium, γδT cells were collected on culture day 14 and resuspended in RPMI-1640 plus 10% FBS in 48-well plates for further culture in the absence of cytokines. Cells were then collected for apoptosis analysis 0 h, 24 h, 48 h, 72 h, 96 h post-IL-2 and/or IL-15 withdrawal. In brief, γδT cells were washed with PBS and resuspended in cell staining buffer (PBS + 3% FBS), followed by Fluorescein isothiocyanate-TCRγδ antibody (BioLegend, San Diego, California, USA) staining at RT for 20 min in dark. Cells were then subjected to a washing protocol with Annexin V-binding buffer (0.1 M Tris (PH 7.4), 1.5 M NaCl, 25 mM CaCl_2_) followed with APC- Annexin V (BioLegend, San Diego, California, USA) staining for 15 min at RT in dark. 7-ADD (BioLegend, San Diego, California, USA) was added to the cell suspension and mixed gently before flow cytometry analysis. Cell events were acquired with a BD FACSCalibur flow cytometer. Data were analyzed with Flowjo software.

### MMP assay

The JC-1 detection assay was performed to measure the MMP of cultured γδT cells after IL-2 and/or IL-15 withdrawal as described above. In brief, cultured γδT cells in the absence of cytokines for 72 h were collected and stained with APC- TCRγδ, the cells were then washed twice with PBS and resuspended with 1 ml of pre-warmed medium containing 2.5 μg/ml JC-1 following incubation at 37 °C in dark for 30 min. The JC-1 fluorescence shift by flow cytometry was used to evaluate the cell MMP change.

### Western blotting

Cultured γδT cells in vitro after cytokine removal were collected by centrifugation at indicated time point within a 72-h culture. The cell pellet was lysed by RIPA buffer with protease inhibitor cocktail (Roche Molecular Biochemicals, Mannheim, Germany) and 1% PhosSTOP (Roche, Basel, Switzerland) with three times freeze (−80 °C) and thaw RT cycles. The lysate was centrifuged at 12000 rpm for 5 min at 4 °C and the supernatant was harvested as a total protein sample. Protein concentration was determined by a Pierce™ BCA Protein Assay Kit (Thermo Scientific, USA) following the manufacturer’s instructions. The protein was separated by 12% SDS-PAGE before being transferred to a methanol pre-soaked PVDF membrane (Millipore, Bedford, MA, USA). The membrane was blocked with 5% skimmed milk in Tris-buffered saline with 0.1% Tween 20 detergent (TBST)-buffer (50 mM Tris-HCl (pH 7.4), 0.9% NaCl, and 0.1% Tween 20) for 2 h at RT followed by an overnight incubation at 4 ºC with primary antibodies: anti-Mcl-1 antibody (cat. no. D2616) from Santa Cruz Biotechnology; anti-Bcl-2 antibody (cat. no. GR3232704-8) from Abcam; anti-ERK1/2 antibody (cat. no. 21), anti-P-ERK antibody (cat. no. 18), anti-STAT5 antibody (cat. no. 1), anti-P-STAT5 antibody (cat. no. 9) and anti-β-actin antibody from Cell Signaling Technology. The signal was detected with an ECL detection reagent after 1 h incubation with horseradish peroxidase (HRP)-conjugated secondary antibody. Signals on immunoblots were quantified using ImageJ software.

### Cytotoxicity assays

To measure the tumoricidal capacity of in vitro expanded γδT cells in different cytokine-conditioned culture mediums, cytotoxicity assays were done with a CytoTox96^®^ Non-Radioactive Cytotoxicity Assay kit (Promega Corporation, Madison, WI, USA) according to the manufacturer’s protocol. NB cell line SH-SY5Y was used as target cell. In brief, a total of 2 × 10^5^ γδT cells were seeded in a round-bottom 96-well culture plate as the spontaneous LDH release control for effector cells; a total of 2 × 10^4^ SH-SY5Y cells were seeded as the spontaneous LDH release control and equal numbers were also seeded as the maximal LDH release for target cells; effect cells were incubated with target cells at the ratio of the effector: target with 10:1 as the experimental well. After 5 h incubation at 37 °C, the lysis solution was added to the target cell control to determine maximal LDH release. Then a total of 50 µl/well lysis supernatant was transferred to a flat-bottom 96-well plate with 50 µl/well-reconstituted substrate mix. Following incubation in dark for 30 min, 50 µl stop solution was added and absorbance was measured at 490 nm by TriStar^2^ LB 942 Multimode Reader (Berthold, Germany). The cytotoxicity of effector γδT cells to target cells was calculated.

### Flow cytometry

For surface staining, the PBMCs and cultured γδT cells were stained with the indicated antibodies (Anti-human CD3, CD25, CD215, TCRγδ, CD56, CD69, HLA-DR) at RT for 20 min in dark. For cytotoxic molecules detection, γδT cells were treated with 50 ng/ml PMA, 1 ng/ml ionomycin, and GolgiStop protein transport inhibitor (BD Biosciences, San Jose, CA, USA) for 5 h. Intracellular staining with antibodies (Anti-human IFN-γ, Granzyme B, perforin) was carried out with a Fixation/Permeabilization Solution Kit (BD Biosciences, San Jose, CA, USA) according to the manufacturer’s protocol. All antibodies were from BioLegend (San Diego, CA, USA) and titration of best concentration was determined before experiments. Cell events were acquired using a BD LSRFortessa X20 flow cytometer, and the collected data were analyzed using FlowJo v10.

### RT-PCR

Total RNA from PBMCs and NB tissues was purified using the Direct-zol RNA Miniprep (ZYMO research, Orange, CA, USA), and the concentration was determined by a NanoDrop ND-8000 (Thermo Fisher Scientific Inc., Waltham, MA, USA). Reverse transcription was performed according to standard protocols using a RevertAid First Strand cDNA Synthesis Kit (Thermo Fisher Scientific Inc., Waltham, MA, USA). qPCR was performed using SYBR Green PCR Master Mix (TIANGEN, Beijing, China) and the fluorescence was read by a QuantStudio 6 flex real-time PCR system (Applied Biosystems, Foster City, CA, USA). Relative expression was calculated by the 2^ΔΔCt^ method with GAPDH as the endogenous housekeeping gene control.

PCR primer sequences:

**Table:** 

Gene	Forward 5′–3′	Reverse 5′–3′
GAPDH	GGAGCCAAAAGGGTCATCACTC	GAGGGGCCATCCACAGTCTTCT
IL-15	GGCCCAAAGCACCTAACCTA	AGGAAGCCCTGCACTGAAAC
IL-15Rα	TGGCTATCTCCACGTCCACTGT	CATGGCTTCCATTTCAACGCTGG

### Measurement of IL-15

Plasma concentration of IL-15 was quantified with the ELISA kit (BioLegend, San Diego, CA, USA) following the manufacturer’s instructions. Briefly, 100 μl serum of each sample or standard was added to the wells pre-coated with capture antibody. Then 50 μl Biotin-conjugated detecting antibody was added and incubated at RT for 2 h. The plate was then washed and 100 μl streptavidin-HRP added to all wells for 1 h incubation at RT. Following with plate wash, 100 μl substrate was added and incubated at RT for 30 min in dark. The reaction was stopped with stop solution and colorimetric signals were collected by an Elisa reader (Berthold, Germany) at OD 450.

### Statistical analysis

The student’s *t* test (paired or unpaired) was used for statistical analysis between two groups. Multiple group comparisons were performed by one-way analysis of variance (ANOVA) with the appropriate post hoc test. Data were represented as mean ± standard error of the mean. Statistical analysis was performed using GraphPad Prism software (GraphPad Software, San Diego, CA, USA), and statistical significance was denoted as *, p < 0.05; **, *p* < 0.01 and ***, *p* < 0.001.

## Supplementary information

Supplementary legend

Original Data File

Figure S1

## Data Availability

The data sets used and/or analyzed during the current study are available from the corresponding author on reasonable request.
