# Supplementary material for: Interleukin-15 enhanced the survival of human γδT cells by regulating the expression of Mcl-1 in neuroblastoma
Source: Cell Death Discov. 2022 Mar 29;8:139. doi: 10.1038/s41420-022-00942-5 (PMC8964681; doi:10.1038/s41420-022-00942-5)
Supplement: Supplementary file 1 — Supplementary legend [file 41420_2022_942_MOESM1_ESM.docx]

Supplementary legend

Fig.S1. (A) Representative gating strategy for the flow-cytometric analysis. At culture day 0, 4, 7, 9 and 14, the absolute numbers of γδT cells from HC and NB were measured by flow cytometry.
