# Supplementary figures and images for: Interleukin-15 enhanced the survival of human γδT cells by regulating the expression of Mcl-1 in neuroblastoma
Source: Cell Death Discov. 2022 Mar 29;8:139. doi: 10.1038/s41420-022-00942-5 (PMC8964681; doi:10.1038/s41420-022-00942-5)

**Fig.4B**

β-actin


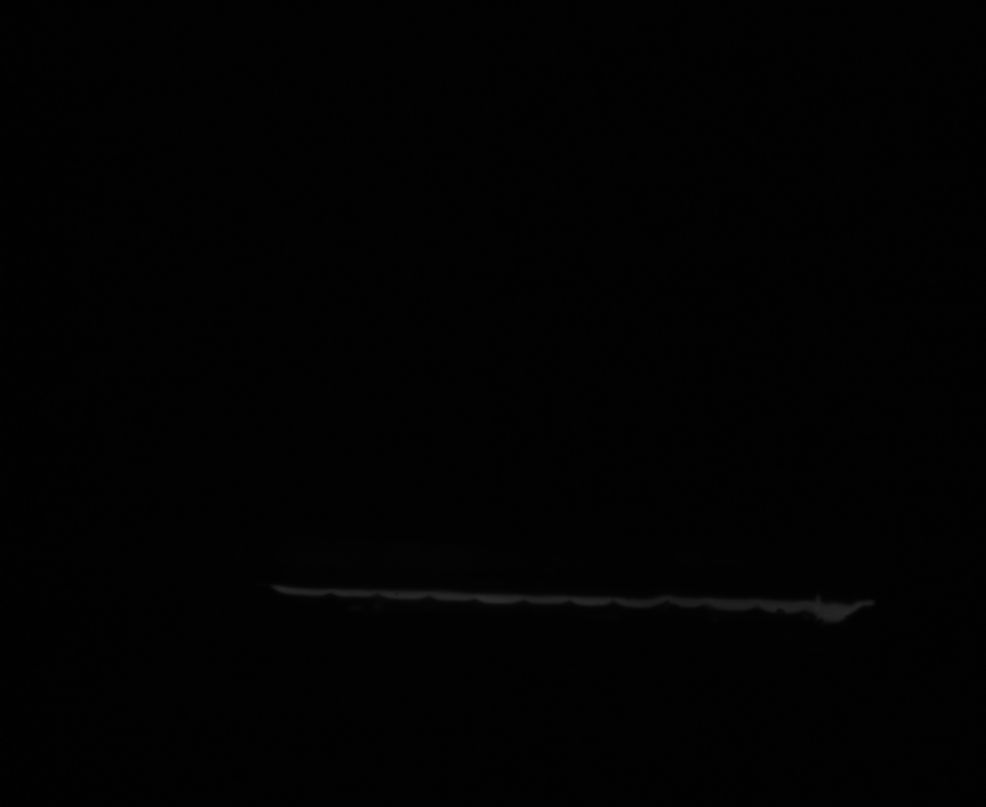


Bcl-2


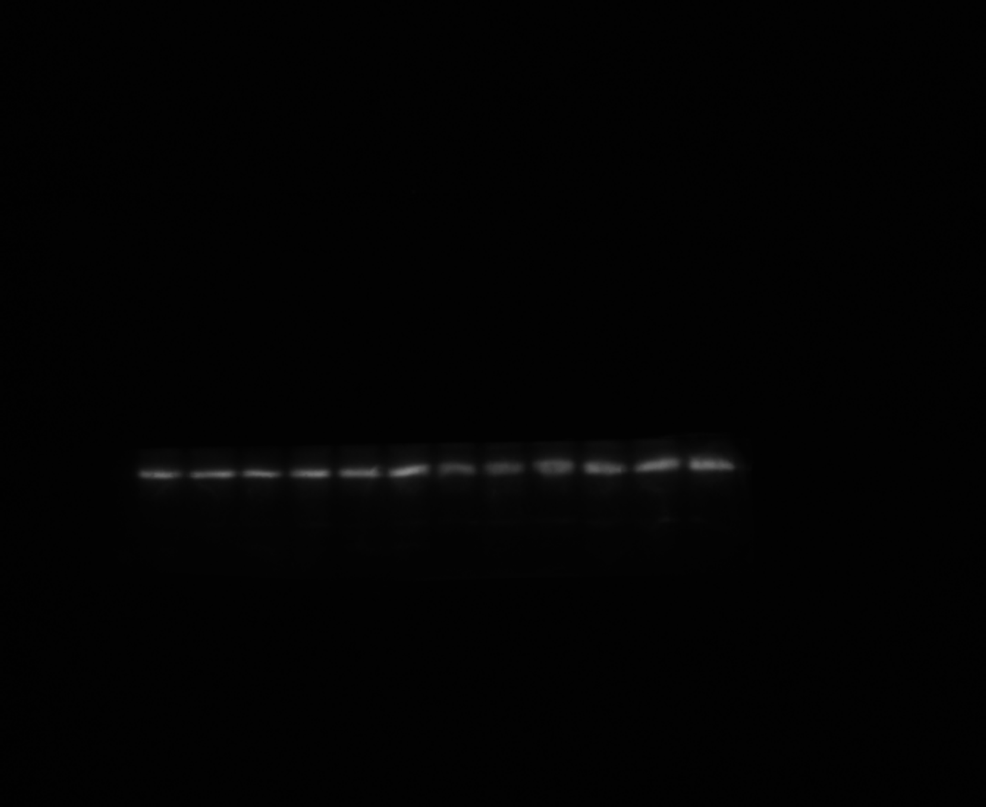


Mcl-1


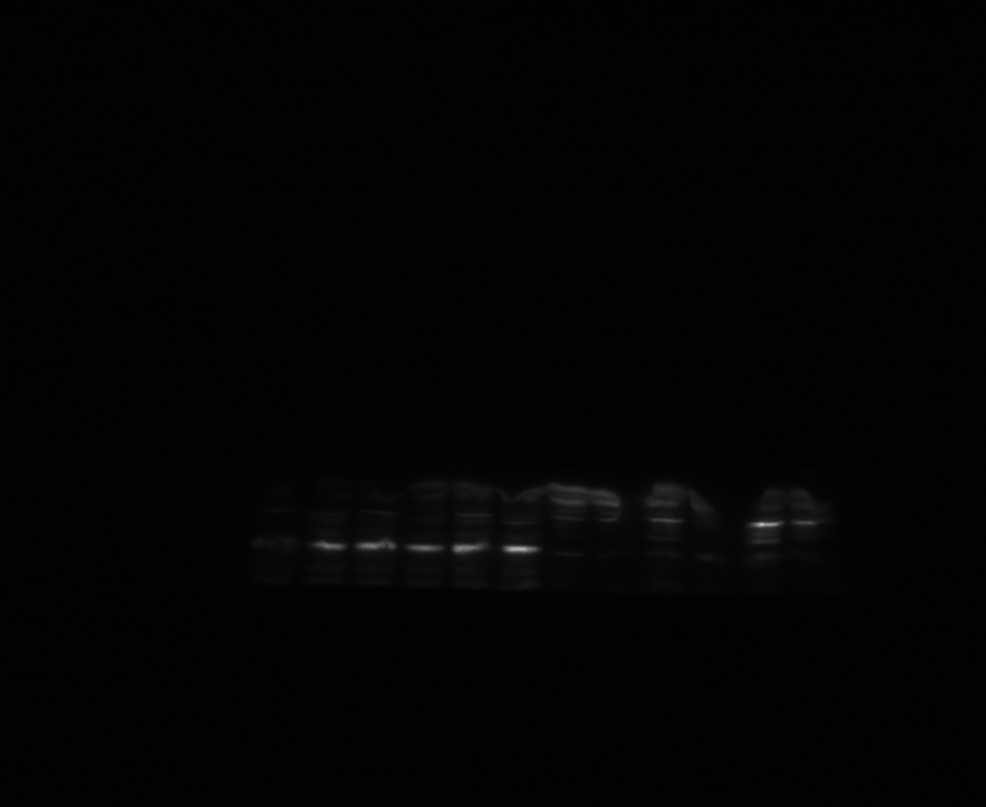


**Fig.4C**

β-actin


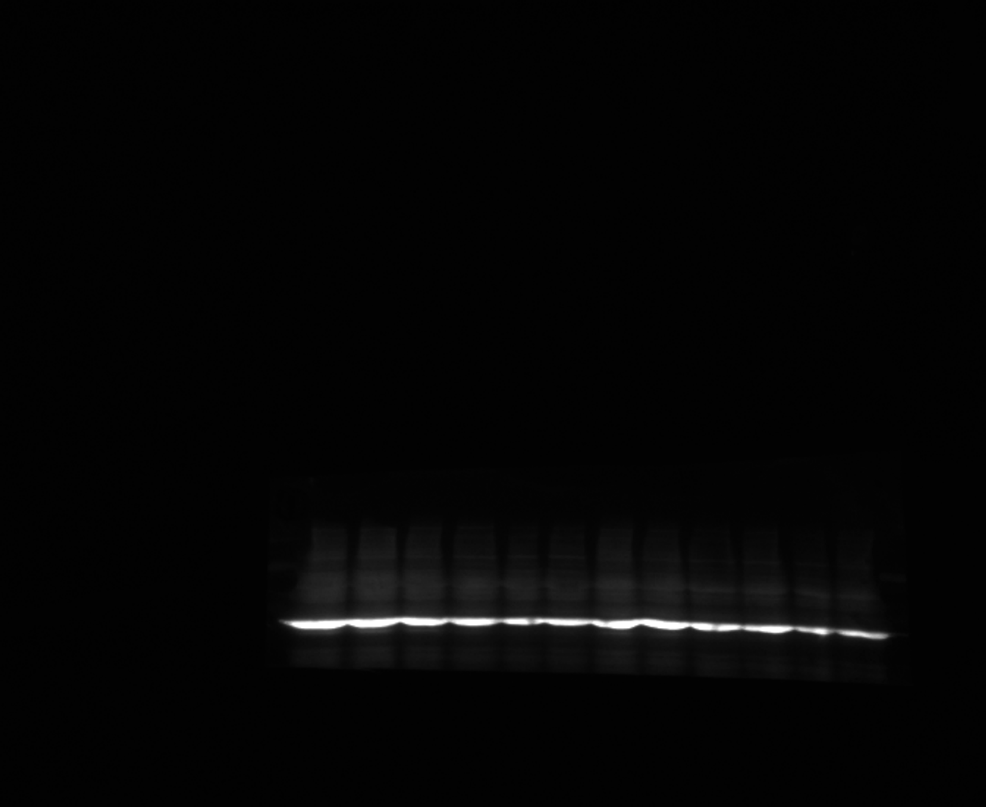


Mcl-1


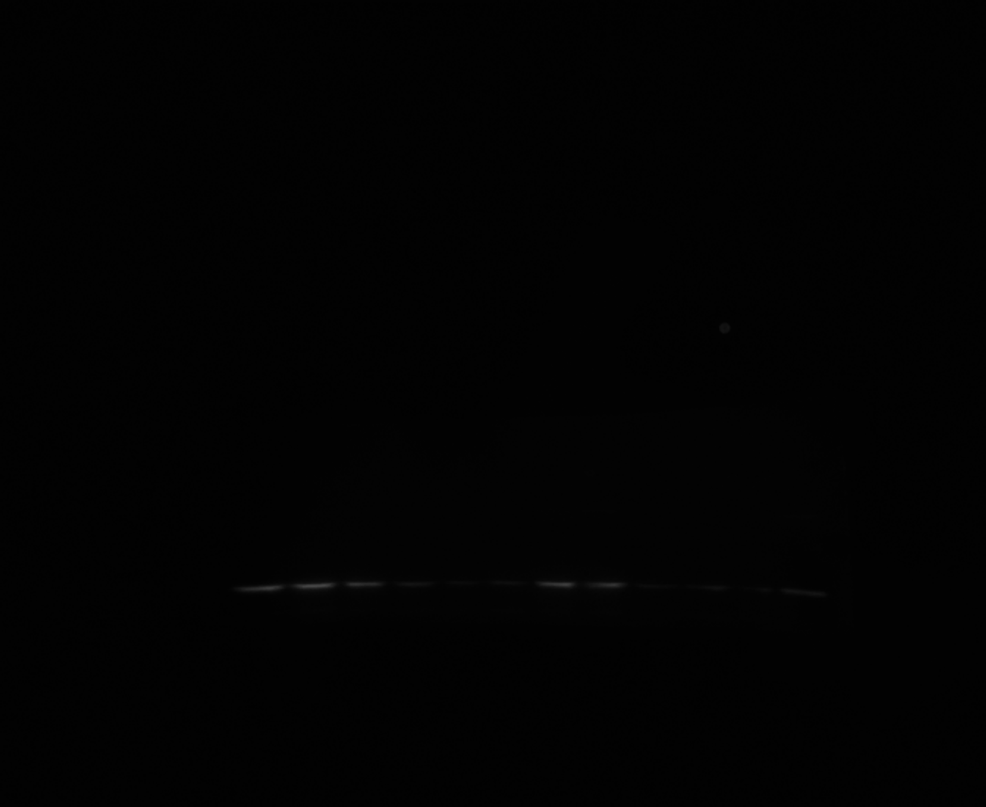


Bcl-2 M1 and M2


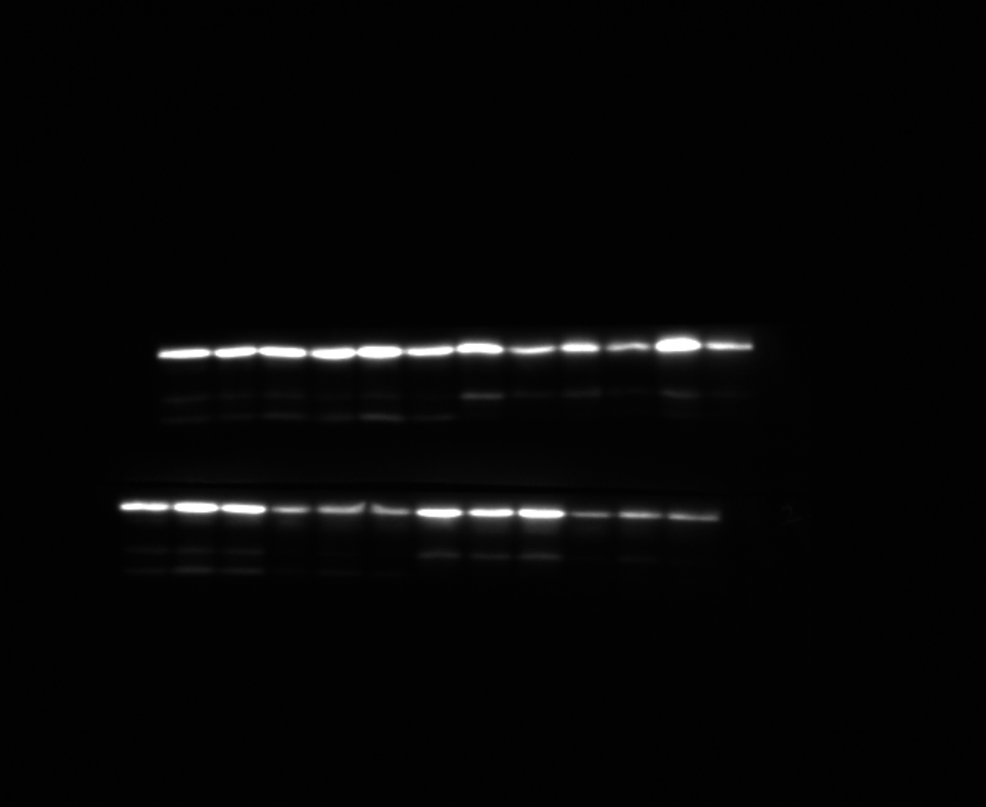


**Fig.4F**

T-ERK


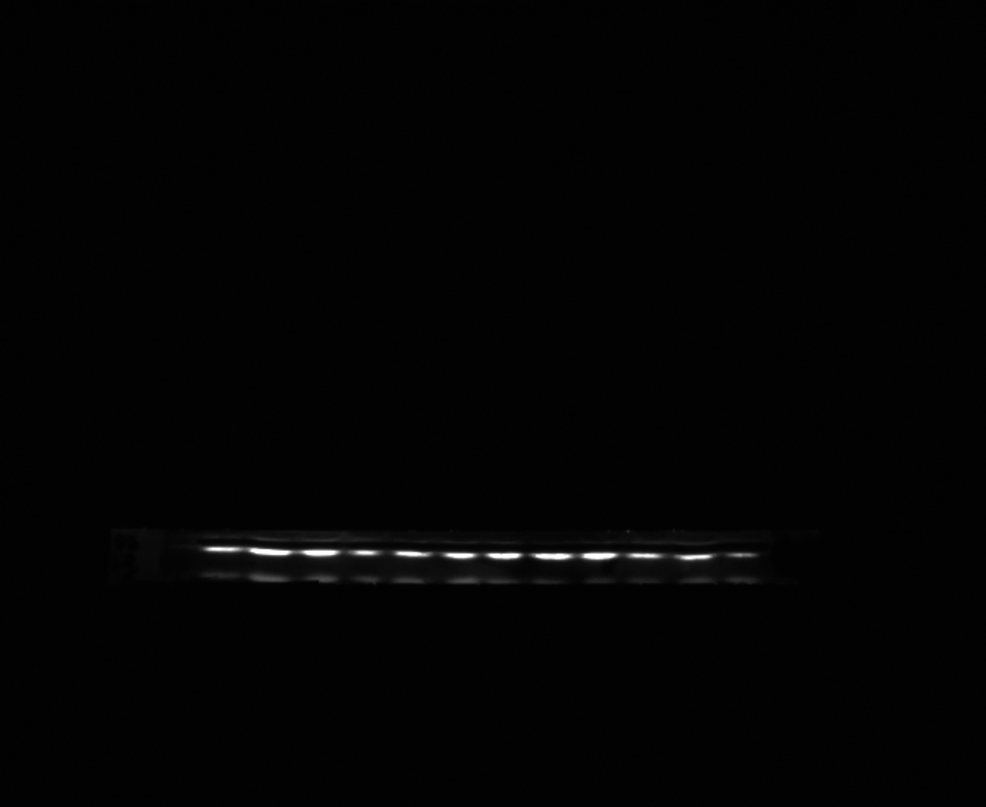


**T-STAT5**


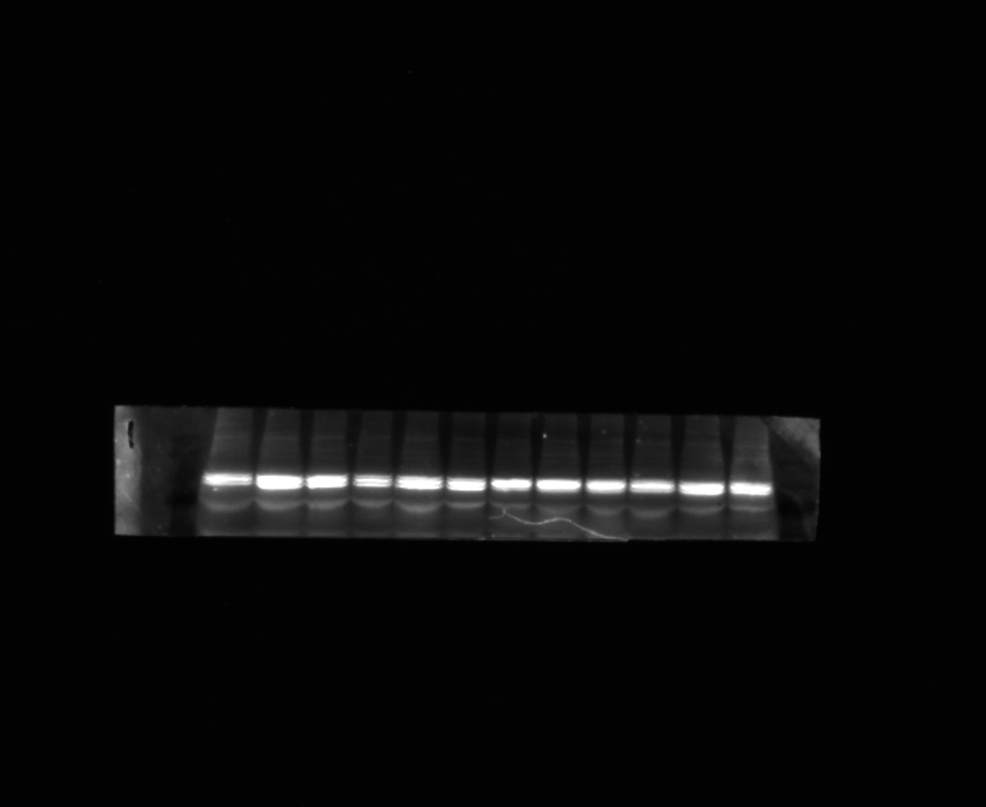


P-STAT5 M1 and M2


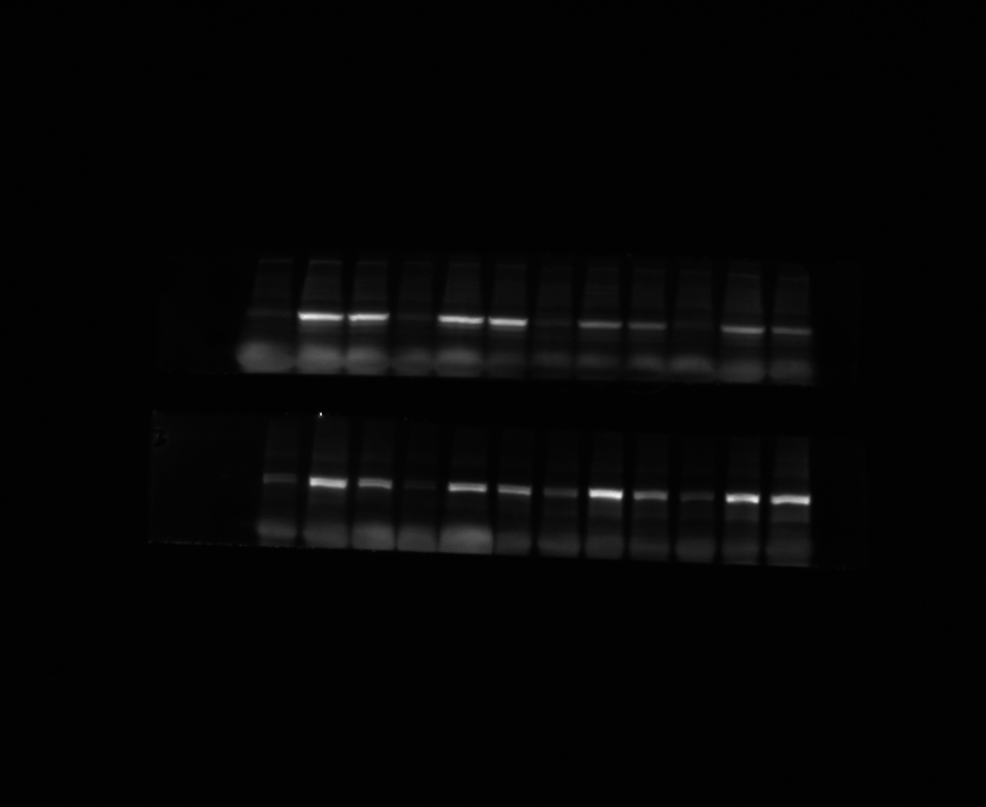


P-ERK


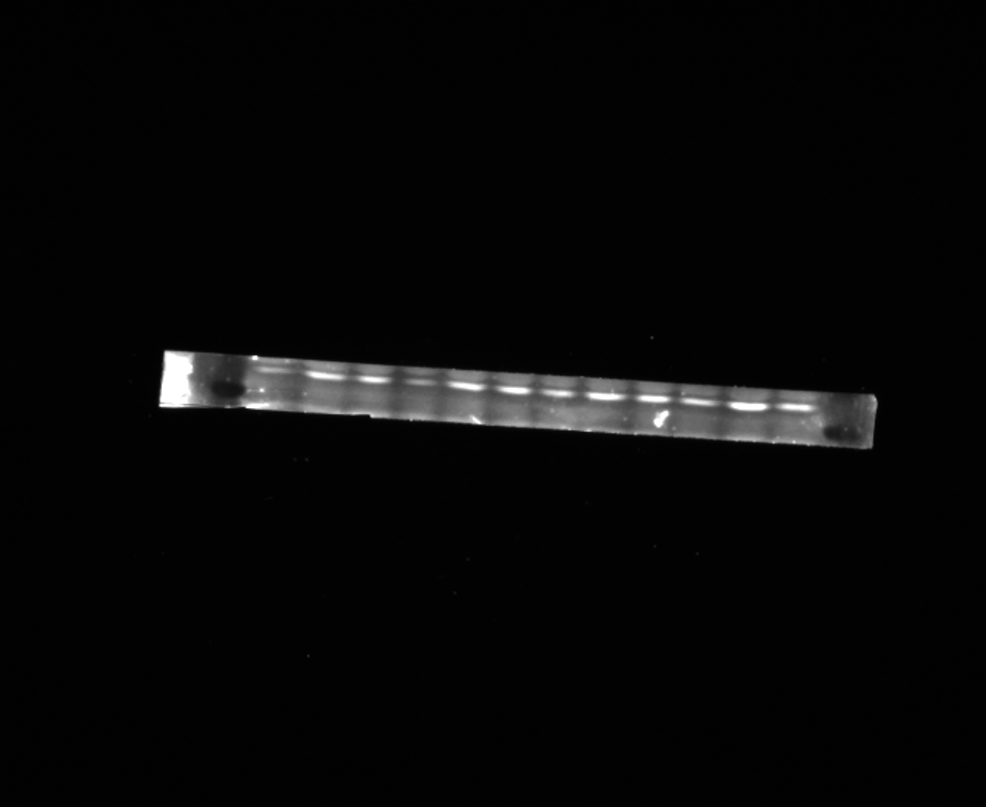


β-actin


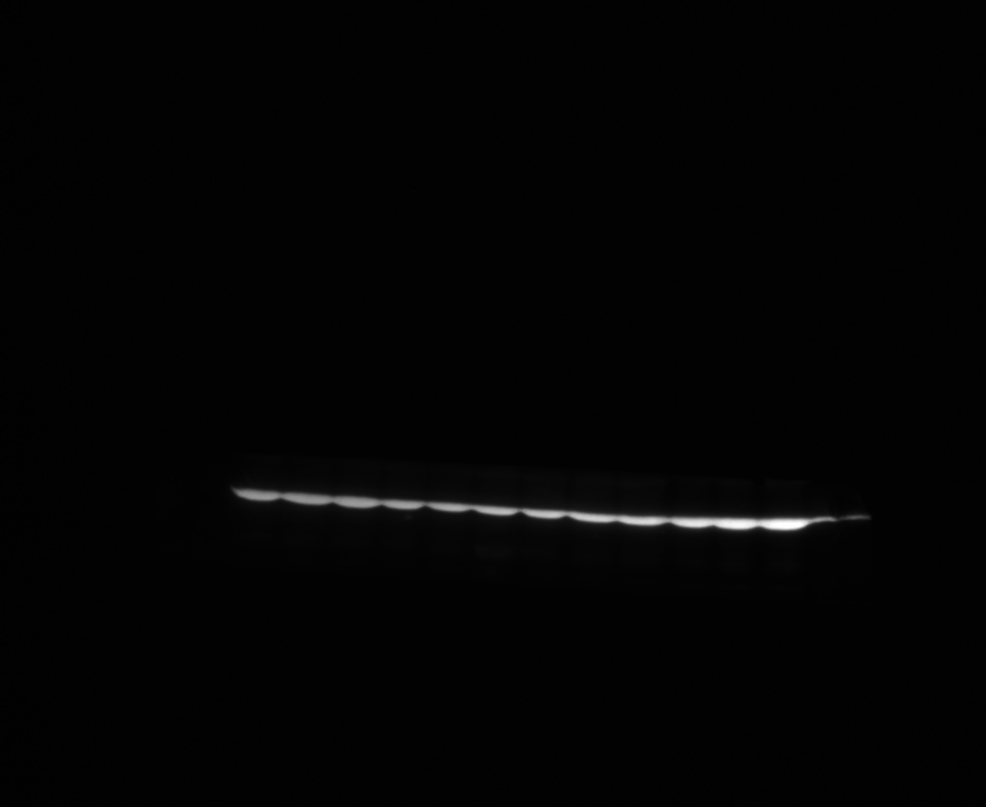

Supplement: Supplementary file 2 — Original Data File [file 41420_2022_942_MOESM2_ESM.docx]

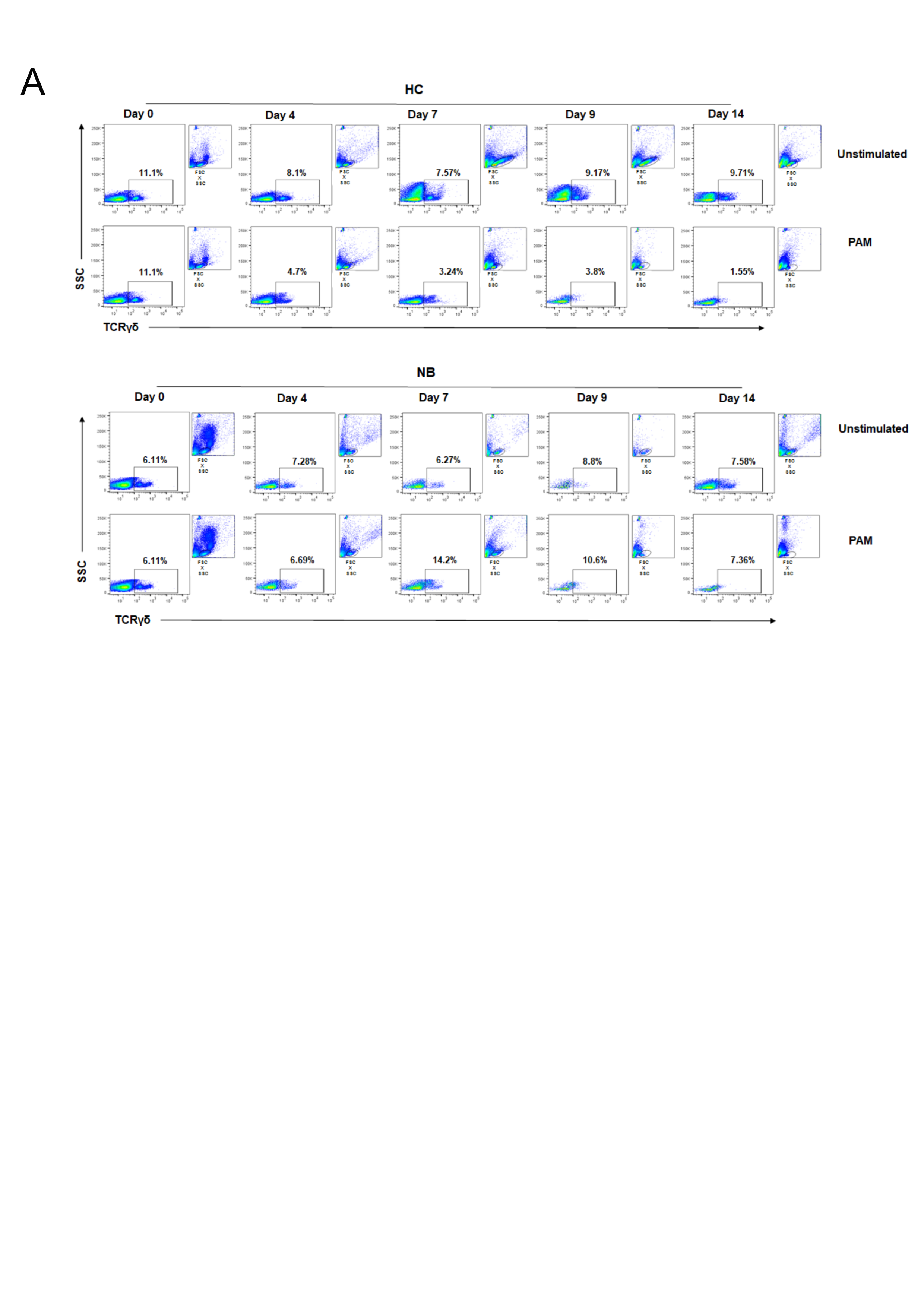

Supplement: Supplementary file 3 — Figure S1 [file 41420_2022_942_MOESM3_ESM.png]
